# Artificial Intelligence in Scientific Judgment: Assistance, Dependence, or Disruption?

**DOI:** 10.12669/pjms.42.6.19570

**Published:** 2026-06

**Authors:** Mariyah Hidayat

**Affiliations:** Dr. Mariyah Hidayat Professor of Anatomy, University College of Medicine and Dentistry, University of Lahore, Lahore, Pakistan. Email: mariyah.hidayat@ucm.uol.edu.pk

**Keywords:** Artificial intelligence, Editorial judgment, Peer review, Scientific critique, Scholarly publishing

## Abstract

Artificial intelligence (AI) is transforming scholarly publishing not by replacing editors or reviewers, but by reshaping how scientific judgment is formed within editorial and peer review systems. Traditionally, manuscripts entered peer review carrying their methodological weaknesses, analytical inconsistencies, and unsupported conclusions for human reviewers to identify and challenge. That sequence is changing. AI is increasingly being used during editorial triage and peer review workflows to evaluate methodological coherence, identify analytical gaps, summarize manuscripts, and highlight potential weaknesses before independent human critique fully develops. While this may improve efficiency, manuscript quality, and the precision of editorial screening, its deeper influence lies in the less visible ways scientific evaluation may become framed by machine-generated interpretation. As reviewers increasingly begin their assessment through AI-generated summaries and critiques, concerns arise regarding the gradual outsourcing of scientific judgment and the potential erosion of independent critical thought. These shifts are emerging within academic systems already strained by publication pressure, reviewer fatigue, and expanding research output. AI may strengthen scholarly publishing by supporting human evaluation, but its growing influence also raises an important question: can scientific judgment remain truly independent when machine interpretation increasingly precedes human critique?

## INTRODUCTION

Peer review was once the first place where a manuscript faced critique, but that is no longer always the case. Reviewers questioned its assumptions, examined its methods, and brought its conclusions back to what the evidence could support. Peer review was never meant to be comfortable. Its purpose was to expose weakness before publication. That sequence is now changing. AI is increasingly becoming the first critical reader of a manuscript, directing editors’ attention to specific strengths and weaknesses before peer review begins.^1^ That influence may sharpen human critical appraisal, but it may also constrain the independent judgment of editors and reviewers. Organizations such as the Committee on Publication Ethics, the International Committee of Medical Journal Editors, and major scientific publishers have already begun issuing guidance on the responsible use of AI in manuscript handling, reflecting the speed with which these tools are entering scholarly workflows.^2^

### How Editorial Judgment Begins Earlier:

Until recently, editorial triage was largely procedural, covering scope, ethics, formatting, and completeness. It prepared manuscripts for peer review, but it did not question their scientific reasoning. That responsibility belonged to reviewers. Artificial intelligence has expanded what editorial triage can do. What was once a procedural checkpoint has become an analytical one. Today, a manuscript may meet its first critical challenge during editorial triage through artificial intelligence, long before peer review begins. What once depended largely on reviewer-stage critique can now begin during the earliest editorial screening process. The editorial desk is beginning to evolve from a gateway of procedural checks into a space where deeper scientific inconsistencies can be identified earlier. This does not replace peer review or turn editors into subject experts, but it shifts editorial engagement from procedural screening to early scientific evaluation, changing where scientific judgment begins.^3^

### How AI Frames Peer Review:

AI is changing not only what enters peer review, but the process through which peer review itself begins. Reviewers now receive manuscripts that are often stronger in structure and clearer in presentation, allowing greater attention to scientific novelty, relevance, and contribution to knowledge. AI is also entering the reviewer’s own workflow, where it is increasingly used to summarize manuscripts, identify methodological weaknesses, and highlight analytical inconsistencies before critique begins.^4^ While this may improve efficiency, it also changes the starting point of evaluation. What the machine identifies first often becomes the reviewer’s first point of attention, and in scientific critique, starting points matter. The first question often shapes the questions that follow. It may begin to influence not only the review process, but potentially the fate of the manuscript itself. What begins as assistance can quietly become direction.

### The Quiet Outsourcing of Judgment:

The real risk is not machine assistance, but the quiet outsourcing of scientific judgment. Scientific critique has traditionally begun with an independent human reading of a manuscript, with the reviewer deciding what appears convincing, what appears weak, and what deserves closer scrutiny. As reviewers increasingly rely on AI-generated summaries before forming their own interpretations, part of the intellectual work of scientific evaluation begins to shift outward.^4^ The framing of concerns, identification of weaknesses, and direction of criticism may increasingly emerge from machine-generated interpretation rather than independent human judgment. Instead of constructing an independent reading of a manuscript, reviewers may begin with a machine-generated interpretation and build their critique around it. The human still makes the final decision, but the intellectual framing of evaluation may already have been shaped elsewhere. Over time, this may not weaken peer review, but it may change the way scientific judgment is formed, making it less independent and less original. Science has always depended on originality of thought before originality of findings.

### A Question for Academic Systems:

The speed with which artificial intelligence has entered editorial systems tells us something important. Scholarly publishing was already under strain. Across academic systems worldwide, publication has become increasingly tied to promotion, career progression, funding, rankings, and institutional recognition.^5^ Research output continues to expand, while the human capacity to critically review it has not grown at the same pace.^6^ As scientific production accelerates, the burden on peer review becomes heavier.

Artificial intelligence did not create this pressure. It entered because academic systems had already normalized a volume of scientific production that human review struggles to sustain. The deeper question lies within the system itself. Have institutions and policy frameworks accelerated research production beyond the human capacity to critically evaluate it? If so, AI is not simply assisting scholarly publishing. It is compensating for a system stretched beyond its own limits.

## CONCLUSION

Artificial intelligence is changing scholarly publishing not by replacing human judgment, but by arriving before it. That may be both its greatest strength and its greatest influence. But science has never relied on efficiency alone. It has relied on human doubt, the discipline of questioning what appears convincing and challenging what appears complete. AI can strengthen that process, but it must not replace where it begins. The future of scholarly publishing will not be shaped simply by how intelligently machines can read science, but by whether human minds still retain the ability to question it first. Editorial systems must adopt AI in ways that strengthen human judgment without displacing its independence.

### AI Use Disclosure:

ChatGPT is used solely for language refinement and readability enhancement. All concepts, interpretations, arguments, and intellectual content presented in this manuscript remain entirely author generated.
